# Selection of the cutoff value of the tuberculin skin test for diagnosing students who need preventive treatment: A school-based cross-sectional study

**DOI:** 10.3389/fcimb.2022.972484

**Published:** 2022-10-13

**Authors:** Peng Lu, Xiaoyan Ding, Jiansheng Sun, Rong Wang, Jiasong Liu, Qiao Liu, Limei Zhu, Wei Lu

**Affiliations:** ^1^ Department of Chronic Communicable Disease, Center for Disease Control and Prevention of Jiangsu Province, Nanjing, China; ^2^ Department of Chronic Communicable Disease, Center for Disease Control and Prevention of Zhouxu City, Nanjing, China; ^3^ Department of Chronic Communicable Disease, Center for Disease Control and Prevention of Nanjing City, Nanjing, China; ^4^ Department of Chronic Communicable Disease, Center for Disease Control and Prevention of Huaian City, Huaian, China

**Keywords:** tuberculosis, school, outbreak, tuberculin skin test, interferon-gamma release assay

## Abstract

**Objective:**

Tuberculosis outbreaks in schools are common in China. This study aimed to introduce and evaluate a new screening process to help control outbreaks.

**Methods:**

Screening information of students in three schools with tuberculosis outbreaks was collected. QuantiFERON-TB gold in-tube (QFT) results were used as the reference standard to determine the cutoff value of the tuberculin skin test (TST) for diagnosing students who need to have preventive medication.

**Results:**

A total of 1,232 students and teachers from three different schools that all had more than three student patients with tuberculosis were included in this study. In total, 308 (25.0%) students had an induration diameter ≥10 mm; among students in a class different from the index case, the infection rate was 24.4% (264/1,084), which was lower than the rate among students in the same class (29.7%) (P = 0.157). Students in the same class as the index tuberculosis case had a much higher QFT positivity compared to those in a different class (58.1% vs. 7.7%, P < 0.0001). Diagnostic agreement between TST ≥10 mm and QFT was 36.6%. The diagnostic value reached the highest when the induration diameter of TST was ≥9 mm, with a sensitivity and specificity of 94.1% (95% CI: 89.4%–97.1%) and 27.6% (95% CI: 24.9%–30.4%), respectively. The area under the curve (AUC) was 0.664 (95% CI: 0.637–0.690, P < 0.0001).

**Conclusion:**

In tuberculosis outbreaks in schools, if there are three or more cases of students with tuberculosis in a class or if the moderate or strong TST positivity rate is much higher than the normal range in the region, attention should be paid to those with moderately positive TST results. Interferon-gamma release assays (IGRAs) are recommended to be conducted following TST on the day of reading the results, especially among students sharing the same class with the index case. In resource-poor areas lacking IGRAs, the induration diameter of TST can be appropriately reduced from 15 to 9 mm to enhance the sensitivity of TST.

## Introduction

The World Health Organization (WHO) aims to eliminate tuberculosis by 2035, which means a 90% reduction in tuberculosis incidence (<10/100,000) and a 95% reduction in mortality compared to that in 2015 (World Health Organization., 2014). However, this goal seems unattainable given the current rate of decline in tuberculosis incidence (1%–2% annually) (Chiappini et al., 2012). Therefore, to accelerate the achievement of this goal, the diagnosis, and treatment of latent tuberculosis infection (LTBI) are particularly important (Rangaka et al., 2015). LTBI refers to a state of persistent immune response to *Mycobacterium tuberculosis* (MTB) antigens without clinical evidence of manifested active tuberculosis (Mack et al., 2009). About a quarter of the world’s population is currently infected with MTB, which could be a huge reservoir of future tuberculosis cases (Houben and Dodd, 2016). At present, the population having preventive therapy for LTBI in China is mainly students who are in close contact with confirmed active tuberculosis patients in a tuberculosis outbreak in a school and who have a tuberculin skin test (TST) induration diameter ≥15 mm or are accompanied by vesicular lymphangitis or strongly positive TST results or a positive interferon-gamma release assay (IGRA) test (National Health Commission, 2020). Unlike other countries where the TST cutoff value to suggest preventive treatment is 5 or 10 mm, China uses 15 mm as the cutoff value (World Health Organization, 2015). However, for students with TST induration diameters between 5 and 15 mm, nothing is done except review their chest X-ray results after 3 months (National Health Commission, 2020). More and more attention is paid to this group of students due to the high risk of tuberculosis (Lu et al., 2021). At this time, if the positive threshold is also set at 15 mm, it is possible to miss several students who truly need preventive medication, resulting in the further spread of tuberculosis outbreaks in schools. Therefore, how to improve the sensitivity of TST to identify recent LTBI in students is particularly important. This study aimed to improve the sensitivity by adjusting the positive threshold of TST using the IGRA test as the gold standard in three schools where tuberculosis outbreaks had occurred.

## Materials and methods

We conducted a school-based cross-sectional study in eastern China in three schools where tuberculosis outbreaks had occurred. There were more than three student-patients with tuberculosis, and the strongly positive rate of purified protein derivative (PPD) was more than 10% in the same class as the index tuberculosis case in all three outbreaks. Among the three outbreaks, two came from a university and one was from a middle school. All contacts had both the TST and QuantiFERON-TB gold in-tube (QFT, a kind of IGRA) examinations in this study.

### Determination of latent tuberculosis infection in students

LTBI was mainly determined by TST and QFT. The Mantoux method was used for TST of the close-contact students (who shared the same classroom or dormitory with the index case). In this study, 0.1 ml of 5 tuberculin units of PPD was injected on the left forearm (Huebner et al., 1993). The TST results were read by experienced nurses 48 to 72 h after administration. The average of the longitudinal and transverse diameters of PPD was measured as the diameter of induration (Huebner et al., 1993). An induration reaction to TST ≥10 mm was defined as positive (Bugiani et al., 2003). An induration reaction of 10 mm ≤ TST < 15 mm was defined as moderately positive, and ≥15 mm was defined as strongly positive. For QFT, we used a cutoff value >0.35 IU/ml. Students with a positive QFT test or strongly positive TST should have preventive treatment (Lu et al., 2021).

### The screening process for tuberculosis outbreaks in schools

**Figure 1:**
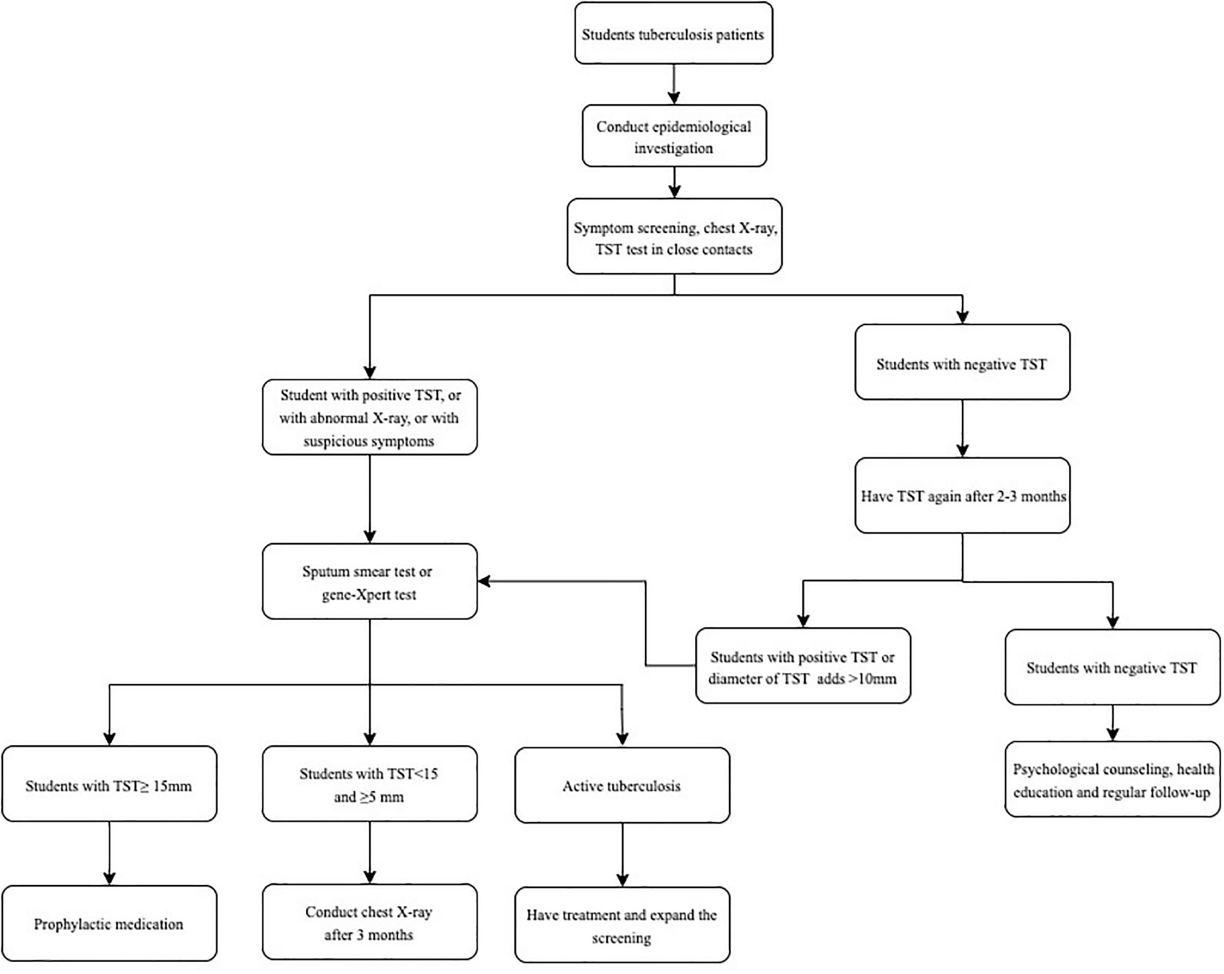
Flowchart of a tuberculosis outbreak in a school dispose. TST, tuberculin skin tests.

After finding the student patients with active tuberculosis, the Center for Disease Control and Prevention (CDC) staff immediately conduct field epidemiological investigations. At first, TST and chest X-rays are performed among close contacts. If one or more new cases of tuberculosis are found in the first screening or a strongly positive rate of the TST detection in close contacts is significantly higher than that in the same age group in the area, the scope of contact screening is expanded to all general contacts. All close contacts of newly detected cases are also recommended for screening. The contact investigation process has been described previously (Lu et al., 2021) and placed in the Appendix (**Figure 1**).

### Statistical analysis

Continuous and categorical variables were summarized using the mean and variance and the standard 2 × 2 contingency tables, respectively. The frequency of categorical variables was compared using Pearson χ^2^ and Fisher’s exact tests, as appropriate. A kappa (κ) statistic was used to evaluate the concordance between TST and QFT. Kappa coefficients were categorized as poor (κ ≤ 0.20), fair (0.20 < κ ≤ 0.40), moderate (0.40 < κ ≤ 0.60), good (0.60 < κ ≤ 0.80), and very good (0.80 < κ ≤ 1.00) (Lu et al., 2021). QFT was used as the reference standard to diagnose students with LTBI who need prophylactic medication, and the receiver operating characteristic (ROC) curve was used to determine the cutoff value of TST for students who need prophylactic medication. A binary univariable logistic regression model was used to evaluate risk factors for QFT positivity. Variables with P < 0.05 in univariable analysis were entered into the multivariable model. P-values included in the final multiple logistic regression were all <0.05. We used SPSS software (version 23.0, IBM Corporation, Armonk, NY) to analyze all databases.

## Results

### Demographic characteristics

A total of 1,232 students and teachers from three different schools were included in this study. Among the 1,232 students and teachers, 148 (12.0%) had the same class as the index case; 420 (34.1%) were men. The mean age of the 1,232 students and teachers was 20.7 years with a standard deviation (SD) of 4.7. In this study, 308 (25.0%) had an induration diameter <10 mm, and among students in a class different from the index case, the rate was 24.4% (264/1,084), which was lower than the rate among students in the same class (44/148, 29.7%) (P = 0.157); however, students in the same class showed a much higher QFT positivity than the students in a different class (58.1% vs. 7.7%, P < 0.0001) (**Table 1**).

**Table 1:** Demographic characteristics of the 1,232 participants overall and those sharing the same class with the index case.

Demographic characteristics	All students (N, %)	Students in a class different from the index case (N, %)	Students in the same classas the index case (N, %)
N	1,232 (100)	1,084 (88.0)	148 (12.0)
Sex			
Male	420 (34.1)	356 (32.8)	64 (43.2)
Female	812 (65.9)	728 (67.2)	84 (56.8)
Age (mean ± SD), years	20.7 ± 4.7	20.8 ± 4.8	20.0 ± 3.8
Induration diameter of PPD (mean ± SD), mm	9.0 ± 4.8	8.9 ± 4.7	9.4 ± 5.7
PPD ≥10 mm			
No	308 (25.0)	264 (24.4)	44 (29.7)
Yes	924 (75.0)	820 (75.6)	104 (70.3)
QFT results			
Negative	1,063 (86.3)	1,001 (92.3)	62 (41.9)
Positive	169 (13.7)	83 (7.7)	86 (58.1)

SD, standard deviation; PPD, purified protein derivative; QFT, QuantiFERON-TB gold in-tube.

### Sensitivity and specificity of the tuberculin skin test

**Figure 2:**
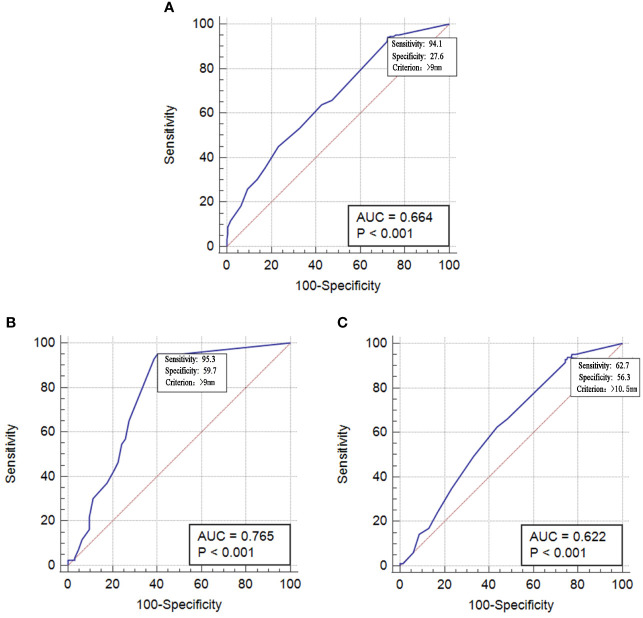
Operating characteristic curve tuberculin of skin test in diagnosing students having preventive treatment. **(A)** All 1232 student. **(B)** 148 students in same class with index case. **(C)** 1084 students in different class with index case.

Among all of the 1,232 individuals, the diagnostic value reached the highest when the induration diameter of TST was ≥9 mm, with a sensitivity and specificity of 94.1% (95% CI: 89.4%–97.1%) and 27.6% (95% CI: 24.9%–30.4%), respectively, and the area under the curve (AUC) was 0.664 (95% CI: 0.637–0.690, P < 0.0001). Among students in the same class as the index case, the cutoff criterion was also ≥9 mm. Aside from a high sensitivity (95.3%, 95% CI: 88.5%–98.7%), a higher specificity was also observed (59.7%, 95% CI: 46.4%–71.9%). However, among students in a class different from the index case, the diagnostic value reached the highest when the induration diameter of TST was ≥10.5 mm, with a sensitivity and specificity of 62.7% (95% CI: 51.3%–73.0%) and 56.3% (95% CI: 53.2%–59.4%), respectively (**Figure 2**).

### Diagnostic agreement of the tuberculin skin test and QuantiFERON-TB gold in-tube test

Among 924 students with TST ≥10 mm, 156 (16.9%) had a negative QFT result, and among 308 students with TST <10 mm, 13 (4.2%) had a positive QFT result (P < 0.001). Diagnostic agreement between TST ≥10 mm and QFT was 36.6% (95% CI: 33.9–39.3) and 30.7% (95% CI: 28.0–33.5) in the different class and 79.7% (95% CI: 73.2–86.3) in the same class as the index case. The kappa coefficient was 0.07 (95% CI: 0.05–0.09) in all individuals, and 0.03 (95% CI: 0.02–0.05) in the different class and 0.57 (95% CI: 0.43–0.69) in the same class.

### Risk factors for QuantiFERON-TB gold in-tube positivity

QFT positivity increased with lower age [odds ratio (OR) = 0.94, 95% CI: 0.89–0.99, P = 0.030], women (OR = 1.44, 95% CI: 1.03–2.01, P = 0.031), a higher induration diameter of PPD (OR7nbsp;= 1.21, 95% CI: 1.14–1.28, P < 0.0001), and being in the same class as the index case (OR = 16.73, 95% CI: 11.26–24.86, P < 0.0001). In the multivariable model, QFT positivity was associated with an increasing induration diameter of PPD [Adjust OR (AOR), 0.2 per 1-mm increase in induration diameter, 95% CI, 0.14–0.27, P < 0.0001] and being in the same class as the index case (AOR = 18.50, 95% CI: 11.90–28.77, P < 0.0001) (**Table 2**).

**Table 2:** Logistic regression analysis of the risk of QuantiFERON-TB gold in-tube positivity.

Characteristic	QuantiFERON-TB gold in-tube positivity		
	(≥0.35 IU/ml)		
	N infected/N total (%)	cOR (95% CI), P-value	aOR (95% CI), P-value
Age (continuous)		0.94 (0.89–0.99), 0.030	0.97 (0.92–1.02), 0.234
Sex			
Male	70/400 (16.7)	Reference	Reference
Female	99/812 (12.2)	1.44 (1.03–2.01), 0.031	1.33 (0.89–1.98), 0.158
Induration diameter of TST (continuous)		1.21 (1.14–1.28), <0.0001	1.20 (1.14–1.27), <0.0001
In the same class as the index case			
No	83/1,084 (7.7)	Reference	Reference
Yes	86/148 (58.1)	16.73 (11.26–24.86), <0.0001	18.50 (11.90–28.77), <0.0001

TST, tuberculin skin test; cOR, crude odds ratio; aOR, adjusted odds ratio.

## Discussion

A tuberculosis outbreak in a school is of particular concern to tuberculosis control in China (Tagarro et al., 2011; Gao and Mei, 2020; Lu et al., 2021). One reason was that students with a strongly positive TST or a positive IGRA found in the early tuberculosis screening process in schools refused to take preventive treatment and continued to attend classes (Lu et al., 2021). Unlike other countries where the induration cutoff value of TST to suggest preventive treatment is 5 or 10 mm, China uses a cutoff of 15 mm (World Health Organization, 2015). This may result in missed diagnoses for students who could benefit from preventive treatment (Lu et al., 2021). In this study, we used concurrent QFT testing to allow us to explore the cutoff criteria for TST. We found that in tuberculosis outbreaks in schools, having three or more active tuberculosis patients in the same class as the index tuberculosis case, the cutoff value of TST should be set to 9–10.5 mm, and if students who were screened shared the same class with the index case, the cutoff criteria can be reduced and set to 9 mm.

TSTs and IGRAs are the two tests recommended by WHO to diagnose LTBI, and individuals with a PPD induration diameter ≥5 or ≥10 mm or an IGRA-positive test are regarded as having LTBI who should have preventive treatment (World Health Organization, 2020). In China, however, 15 mm was the cutoff value of TST to have preventive treatment (National Health Commission, 2020), which may lead to a missed diagnosis of individuals who need preventive treatment. In our previous study of a tuberculosis outbreak in a school, we found nine student-patients with tuberculosis, of whom six had an induration diameter of PPD between 5 and 15 mm (Lu et al., 2021). If the positive threshold of TST for students who need preventive medication is also set at 15 mm, it is possible for some students who need therapy to prevent the ongoing development of tuberculosis disease. Several different thresholds for TST have been adopted in global guidelines to reduce false-positive results of TST to diagnose LTBI (WHO and Guidelines Approved by the Guidelines Review Committee, 2014; Lu et al., 2020). Similarly, we could try to find as many students as possible who need prophylactic medication by reducing the induration diameters of TST. According to Chinese school tuberculosis prevention and control regulations, students with a TST induration diameter ≥15 mm should receive preventive treatment, yet in certain special circumstances, such as when there are three or more tuberculosis patients in a class, or a moderate or a strong TST positivity rate is much higher than the normal range in the region, the focus should be on students with TST induration diameters of 10–15 mm. The reasons are as follows (1): If the above situation occurs, the transmission is not recent, which attaches great importance to the impact that a delay in diagnosis is one of the most important factors behind tuberculosis outbreaks (Phillips et al., 2004; Huang et al., 2016). (2) Both TST and IGRA have a window period (time between individuals are infected and when the immunological response becomes measurable) of up to 8 weeks (Menzies, 1999; Abubakar et al., 2011), which indicates that more time is needed to test the infection status of a case. A premature start of screening would result in misclassification of the LTBI status, which means that students who are recently infected may be incorrectly diagnosed as remotely infected or non-infected.

Recently, a new two-step screening approach to individuals was recommended in some countries such as the United Kingdom, Canada, Italy, Spain, Saudi Arabia, and China, that is, using TST as the first line followed by IGRA as the second step if the TST results were positive (Denkinger et al., 2011; Internal Clinical Guidelines Team (UK), 2016; Schaberg et al., 2017; Jia et al., 2022). Considering the complexity and high cost of IGRA, it is unlikely to be conducted across all student populations. In our study, students in the same class as the index case had a lower cutoff threshold than those in a different class. It is not difficult to understand that students in the same class would have had more contact and longer contact time than others and would have had a much higher risk of tuberculosis (Conditions NCCfC, 2006). This new two-step screening approach would be conducted in key populations such as students in the same class. If conditions do not allow, the cutoff value of TST for having treatment could be set to 9–10 mm for students who are in close contact with the index cases such as those in the same class.

Our study has a limitation in that all of the students took TST first followed by the QFT test, which might overestimate the QFT positivity due to the potential boosting effect of TST on IGRA (van Zyl-Smit et al., 2009). However, most of the students had the QFT test immediately after reading the TST results about 3 days after TST. A study suggested that TST almost had no boosting effect on IGRAs if IGRAs were performed within 3 days of performing TST (van Zyl-Smit et al., 2009). There is also a study that showed that screening for LTBI using TST on the day of reading is a reliable approach, as the specificity of QFT is not affected by prior TST administration (Leyten et al., 2007).

## Conclusion

In conclusion, in tuberculosis outbreaks in schools, if there are three or more tuberculosis cases in students in a class or the moderate or strong TST positivity rate is much higher than the normal range in the region, attention should be paid to those with moderately positive TST results. IGRAs are recommended to be conducted following TST on the day of reading the results, especially among students sharing the same class with the index case. In resource-poor areas lacking IGRAs, the induration diameter of TST can be appropriately reduced from 15 to 9 mm to enhance the sensitivity of TST.

## Data availability statement

The original contributions presented in the study are included in the article/supplementary material. Further inquiries can be directed to the corresponding author.

## Author contributions

WL, PL, and XD conceived the study, analyzed the data, and drafted the manuscript. QL participated in the study design. JS, RW, JL, and LZ implemented the field investigation. All authors contributed to the study and have read and approved the final manuscript.

## Funding

This work was supported by the “Key Scientific Research Project of Jiangsu Commission of Health” (grant numbers: ZD2021052 and ZDA2020022).

## Acknowledgments

The authors are grateful to Leonardo Martinez for his help with the article.

## Conflict of interest

The authors declare that the research was conducted in the absence of any commercial or financial relationships that could be construed as a potential conflict of interest.

The handling editor BX declared a past co-authorship with the author WL.

## Publisher’s note

All claims expressed in this article are solely those of the authors and do not necessarily represent those of their affiliated organizations, or those of the publisher, the editors and the reviewers. Any product that may be evaluated in this article, or claim that may be made by its manufacturer, is not guaranteed or endorsed by the publisher.
